# Heterogeneous nuclear ribonucleoprotein A1 post-transcriptionally regulates Drp1 expression in neuroblastoma cells

**DOI:** 10.1016/j.bbagrm.2015.10.017

**Published:** 2015-12

**Authors:** So Jung Park, Heejin Lee, Doo Shin Jo, Yoon Kyung Jo, Ji Hyun Shin, Han Byeol Kim, Hae Mi Seo, David C. Rubinsztein, Jae-Young Koh, Eun Kyung Lee, Dong-Hyung Cho

**Affiliations:** a Department of Gerontology, Graduate School of East–West Medical Science, Kyung Hee University, Yongin, South Korea; b Department of Biochemistry, College of Medicine, The Catholic University of Korea, Seoul, South Korea; c Department of Medical Genetics, Cambridge Institute for Medical Research, University of Cambridge, Cambridge CB2 0XY, UK; d Department of Neurology, University of Ulsan College of Medicine, Asan Medical Center, Seoul, South Korea

**Keywords:** Mitochondria dynamics, Drp1, hnRNP A1, RNA binding protein

## Abstract

Excessive mitochondrial fission is associated with the pathogenesis of neurodegenerative diseases. Dynamin-related protein 1 (Drp1) possesses specific fission activity in the mitochondria and peroxisomes. Various post-translational modifications of Drp1 are known to modulate complex mitochondrial dynamics. However, the post-transcriptional regulation of Drp1 remains poorly understood. Here, we show that the heterogeneous nuclear ribonucleoprotein A1 (hnRNP A1) regulates Drp1 expression at the post-transcriptional level. hnRNP A1 directly interacts with Drp1 mRNA at its 3′UTR region, and enhances translation potential without affecting mRNA stability. Down-regulation of hnRNP A1 induces mitochondrial elongation by reducing Drp1 expression. Moreover, depletion of hnRNP A1 suppresses 3-NP-mediated mitochondrial fission and dysfunction. In contrast, over-expression of hnRNP A1 promotes mitochondrial fragmentation by increasing Drp1 expression. Additionally, hnRNP A1 significantly exacerbates 3-NP-induced mitochondrial dysfunction and cell death in neuroblastoma cells. Interestingly, treatment with 3-NP induces subcellular translocation of hnRNP A1 from the nucleus to the cytoplasm, which accelerates the increase in Drp1 expression in hnRNP A1 over-expressing cells. Collectively, our findings suggest that hnRNP A1 controls mitochondrial dynamics by post-transcriptional regulation of Drp1.

## Highlights

• hnRNP A1 increases Drp1 expression through the interaction with 3′UTR of Drp1 mRNA.
• Down-regulation of hnRNP A1 increases mitochondrial elongation by reducing drp1 expression.
• Down-regulation of hnRNPA1 inhibits 3-NP-mediated mitochondrial dysfunction.
• Over-expression of hnRNP A1 potentiates 3-NP-mediated mitochondrial dysfunction and cell death.
• Treatment of 3-NP promotes translocation of hnRNP A1 to the cytoplasm and enhances Drp1 expression.

## 1 Introduction

Mitochondria are important organelles involved in the production of energy necessary for cell survival and homeostasis [1], [2]. Mitochondrial morphology depends on the balance between two opposing processes, mitochondrial fission and fusion, collectively termed as mitochondrial dynamics. The disruption of this balance results in mitochondrial dysfunction and aberrations in physiological neuronal functions, such as exon transport, synaptic transmission, calcium homeostasis, and neuronal development [3]. Neurons are especially susceptible to changes in mitochondrial dynamics, such as mitochondrial movement, distribution and function, due to their high-energy demand [4], [5]. Abnormalities in mitochondrial dynamics are implicated in the early events of neurodegenerative diseases such as Huntington's disease (HD), Alzheimer's disease (AD), Parkinson's disease (PD), amyotrophic lateral sclerosis (ALS) and Charcot–Marie–Tooth neuropathy (CMT) [6], [7]. HD is an autosomal dominant condition caused by abnormal expansions in a CAG repeat tract in the huntingtin gene (HTT), resulting in an abnormally long poly-glutamine stretch in the protein which renders it aggregate-prone [8]. The 3-nitropropionic acid (3-NP) toxin acts a mitochondrial respiratory complex II inhibitor, which leads to an HD-like pattern of cell loss in rodents [9]. Expression of mutant HTT and 3-NP treatment trigger both mitochondrial fission and mitochondrial dysfunction, and eventually induce neurotoxicity with disrupted axonal transport and synaptic degeneration [10], [11], [12], [13], [14], [15].

The mitochondrial fission and fusion machineries are continuously controlled by several large GTPase proteins. Mitochondrial fission is mediated by dynamin-related protein 1 (Drp1), a dynamin GTPase protein [16]. Drp1 primarily located in the cytosol is recruited to the mitochondria and interacts with outer membrane anchoring proteins such as Fis1, mitochondria fission factor, mitochondria dynamics protein 49 and 50 kDa [6], [17], [18]. At the constriction sites, Drp1 assembles ring-like complex to promote mitochondrial fragmentation process using GTPase hydrolysis [19]. On the other hand, the mitochondrial fusion is controlled by other large GTPase proteins, mitofusin 1/2 (Mfn1/2) and optic atropy 1 (OPA1) [20]. The dimerization of Mfn1/2 tethers outer mitochondrial membranes (OMM) of adjacent mitochondria. And inner mitochondrial membrane (IMM) fusion is regulated by OPA1 which plays role in maintaining mitochondrial DNA and controlling cristae remodeling [21]. Previous studies have reported that Drp1 activity is regulated by several post-translational modifications, such as phosphorylation, S-nitrosylation, SUMOylation, ubiquitination, and O-GlcNacylation [22], [23], [24], [25], [26]. Although the post-translational regulations of Drp1 are well elucidated, the regulation of Drp1 at the gene expression level is still poorly understood.

Gene expression is regulated at the level of transcription and translation. The post-transcriptional regulation of gene expression underlies many aspects of mammalian physiology and pathology [27], [28]. The two main groups of posttranscriptional regulators, RNA-binding proteins (RBPs) and noncoding RNAs contribute to RNA splicing, transport, mRNA stability, RNA storage as well as translational regulation [29], [30]. These processes are associated with numerous cellular events, including cell proliferation, differentiation, migration and cell death [31]. However, the role of RBPs in the regulation of mitochondrial dynamics has not been addressed to date. In the present study, we identified heterogeneous nuclear ribonucleoprotein A1 (hnRNP A1) as a novel regulator of Drp1 expression. This protein directly interacts with Drp1 mRNA and positively regulates Drp1 expression. Down-regulation of hnRNP A1 was found to suppress both mitochondrial dysfunction and cell death in 3-NP-treated cells, while, the over-expression of hnRNP A1 enhanced Drp1 expression and increased mitochondrial fragmentation. Furthermore, hnRNP A1 potentiated 3-NP-induced mitochondrial fission and cell death. Interestingly, we found that treatment with 3-NP facilitates hnRNP A1 translocation to cytosol and significantly enhances the level of cytoplasmic hnRNP A1 bound *Drp1 mRNA*. Taken together, these results suggest that hnRNP A1 regulates mitochondrial dynamics by binding Drp1 mRNA.

## 2 Materials and methods

### 2.1 Cell culture and measurement of mitochondrial length

Neuroblastoma cells, SK-N-MC cells and SH-SY5Y cells were obtained from the American Type Culture Collection (ATCC, Manassas, VA). Cells were cultured at 37 °C in a 5% CO_2_ incubator and maintained in Dulbecco's modified Eagle's medium containing 10% fetal bovine serum and 1% penicillin/streptomycin (Invitrogen, Carlsbad, CA). In order to generate stably expressing cell lines (SK-N-MC/mito-YFP, SK-N-MC/GFP and SK-N-MC/GFP-hnRNP A1), SK-N-MC cells were transfected with pMito-YFP, pEGFP, and pEGFP-hnRNAP A1 using Lipofectamine 2000 according to the manufacturer's protocol (Invitrogen, Carlsbad, CA). The stable transfectants were selected based on their growth in selection medium containing G418 (1 mg/ml) for 10 days. After single cell dropping, the stably expressing clones were selected under a fluorescence microscope (IX71, Olympus, Japan) and were confirmed by Western blotting. Mitochondrial lengths were measured using the Cell Sense Standards software (Olympus, Hamburg, Germany). The average mitochondrial length was determined after selection of the arbitrary end of mitochondrial filament using the free-hand line selection tool to measure length of individual mitochondria obtained randomly selected 15–20 mitochondria per cell. And images were analyzed and digitized using the Cell Sense Standards software.

### 2.2 Reagents

YFP-fused mito-tracker plasmid (pmito-YFP) was previously described [32]. GFP-fused hnRNP A1 plasmid (pEGFP-hnRNP A1) was purchased from Origene Technologies (Rockville, MD). 3-nitropropionic acid (3-NP) was purchased from Sigma (St. Louis, MO). A mitoTracker® probe and Hoechst 33,342 dye were purchased from Invitrogen (Carlsbad, CA). The validated siRNAs targeting for hnRNP A1 (#1, 5′-GCUCUUCAUUGGAGGGUUG-3′), (#2, 5′-CUACAAUGAUUUUGGGAAUUU-3′) and a negative scrambled siRNA (5′-CCUACGCCACCAAUUUCGU-3′) were purchased from Dharmacon (Thermo Scientific). Previously validated Drp1 siRNA (5′-GAGGUUAUUGAACGACUCA-3′) was synthesized from Bioneer (Daejeon, Korea) [32].

### 2.3 Biotin pull-down assay

In order to synthesize biotinylated transcripts, PCR fragments were prepared using forward primers containing the T7 RNA polymerase promoter sequence (T7, CCAAGCTTCTAATACGACTCACTATAGGGAGA), as listed in Supplementary Table 1. Using the purified PCR products, biotinylated transcripts were obtained by reverse transcription using the MaxiScript T7 kit (Ambion, Waltham, MA) and biotin-CTP (Enzo Life Sciences, Farmingdale, NY) [33]. Protein lysates (120 μg/sample) were incubated with 1 μg of biotin labeled transcripts for 30 min at room temperature, and then, the protein-RNA complexes were isolated using streptavidin-coupled Dynabeads (Invitrogen, Carlsbad, CA). Proteins bound to biotinylated transcripts were assessed by Western blotting.

### 2.4 Ribonucleoprotein immunoprecipitation

Ribonucleoprotein immunoprecipitation assay was previously described [34]. Briefly, whole cell lysates were incubated with anti-hnRNP A1 or control IgG-conjugated protein-A beads at 4 °C for 2 h. After further incubation of immunoprecipitated complex with DNase I and protease K, RNAs were isolated using acidic phenol. cDNAs were synthesized using random hexamers and reverse transcriptase (Toyobo, Osaka, Japan) and relative Drp1 mRNA in immunoprecipitated complexes was determined by real time quantitative PCR (RT-qPCR) with 2 × SYBR green PCR master mix (Kapa Biosystems, Wilmington, MA) and gene-specific primer sets (Supplementary Table 1) using StepOne Plus™ instrument (Life Technologies, Waltham, MA).

### 2.5 Preparation of reporter plasmid and luciferase assay

Luciferase reporter plasmid containing 3′UTR sequence of Drp1 mRNA (2373–3535, 3U1 fragment) were generated by cloning of 3U1 fragment at the 3′UTR region of Renila luciferase gene of psiCHECH2 plasmid (Promega, Madison, WI). SH-SY5Y cells were transfected with the reporter constructs (psiCHECK2 of psiDrp1 3U1) after transfection of siRNAs or plasmids, respectively, and reporter activity was analyzed using Dual Luciferase assay system (Promega). Firefly luciferase activity was used for normalization.

### 2.6 Western blotting and cell fractionation

All lysates were prepared with protein sample buffer (62.5 mM Tris–HCl, pH 6.8, 25% glycerol, 2% SDS, 5% β-mercaptoethanol, 0.01% Bromophenol blue) (BioRad, Hercules, CA). Then the samples separated by SDS-PAGE were transferred to PVDF membrane (BioRad). After blocking with 4% skim milk in TBST (25 mM Tris, 3 mM 140 mM NaCl, 0.05% Tween-20), the membranes were incubated overnight with specific primary antibodies at 4 °C. Anti-Drp1 antibody was from BD (San Jose, CA); anti-hnRNP A1 was from Santa Cruz Biotechnologies (Santa Cruz, CA); anti-cleaved caspase-3 was from Cell Signaling Technology (Danvers, MA); anti-Actin antibody was from Millipore (Temecula, CA). For protein detection, the membranes were incubated with HRP-conjugated secondary antibodies (Pierce, Rockford, IL).

### 2.7 Measurement of mitochondrial membrane potential and ATP level

The loss of mitochondrial membrane potential in the depolarized mitochondria was measured with a unique fluorescent cationic dye, TMRE (Tetramethylrhodamine ethyl ester, Invitrogen, Carlsbad, CA). Cells treated with agents were incubated with TMRE, and the fluorescence intensity was measured using plate reader (PerkinElmer Life Sciences) at excitation and emission wavelengths of (549 and 575). Cellular total ATP level was detected with an ATP bioluminescence detection kit (Promega, Madison, WI) according to the manufacturer's protocol.

### 2.8 Measurement of cytotoxicity

Cytotoxicity was quantitated by measuring lactate dehydrogenase (LDH) release from damaged cells. LDH content was determined using an LDH Cytotoxicity Detection Kit (Takara, San Diego, CA) according to the manufacturer's protocol. Briefly, cells transfected with either siRNA or plasmid were exposed to 3-NP for 24 h. The total LDH activity based on conversion the red formazan was examined using spectrophotometry by measuring its absorbance at 490 nm compared with that of untreated control cells.

### 2.9 Statistical analysis

Data were obtained from least three independent experiments, and presented as means ± S.E.M. Statistical evaluation of the results was performed with one-way ANOVA. Data were considered significant at a value of *p < 0.05.

## 3 Results

### 3.1 hnRNP A1 increases Drp1 expression through the interaction with 3′UTR of Drp1 mRNA

**Fig. 1:**
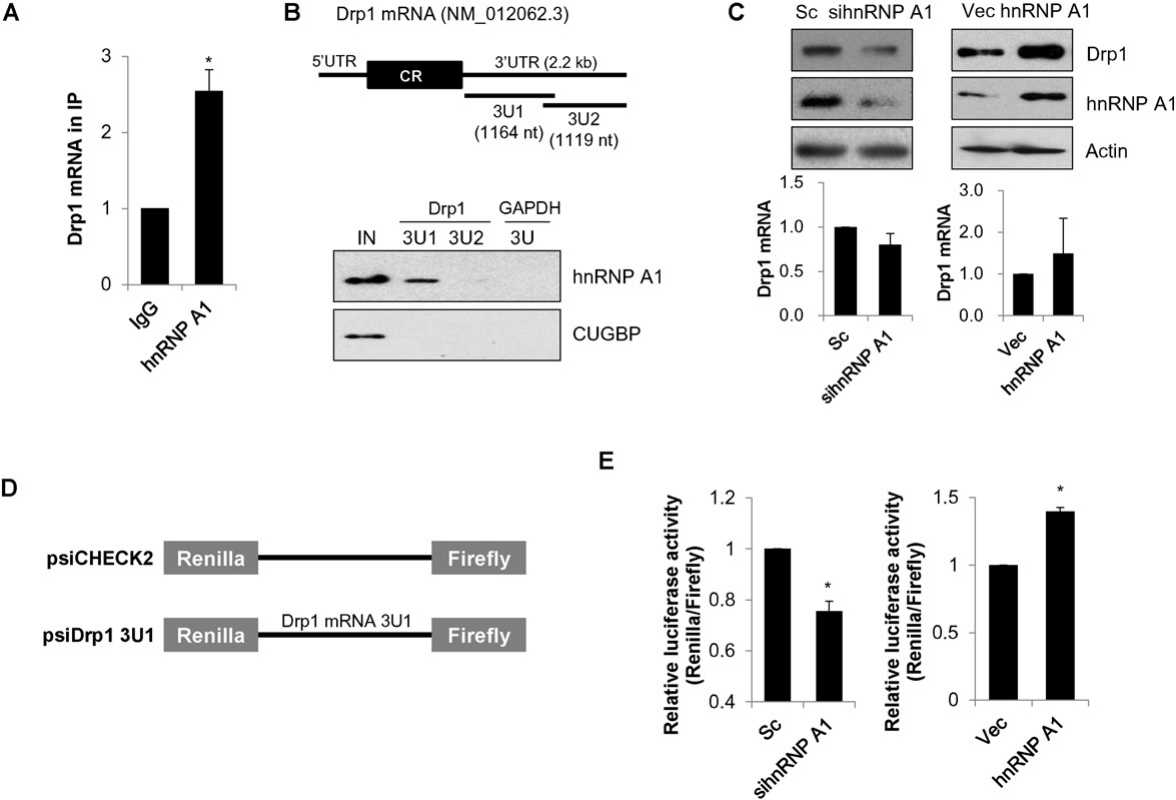
hnRNP A1 regulates Drp1 expression by binding its 3′UTR. (A) SH-SY5Y cell lysates were subjected to RNP-IP complex using anti-hnRNP A1 antibody or control IgG. After isolation of RNAs in the precipitated complex, relative binding of Drp1 mRNA to hnRNP A1 was assessed by RT-qPCR. (B) Schematic description of the Drp1 mRNA (NM_012062) 5′-untranslated region (5′UTR), coding region (CR), 3′-untranslated region (3′UTR). The interaction between hnRNP A1 and biotinylated transcripts of Drp1 mRNA 3′UTR was examined by biotin-pull-down analysis. After incubating biotinylated fragments with cell lysates, the binding was analyzed by Western blotting using hnRNP A1 antibody. GAPDH 3′UTR (GAPDH 3U) was used as a negative control. (C) SH-SY5Y cells were transfected with either hnRNP A1 siRNA (sihnRNP A1, left) or pHA-hnRNP A1 (hnRNP A1, right), and the protein levels were analyzed by Western blotting with indicated antibodies (upper panel) and Drp1 mRNA was measured by RT-qPCR (lower panel). (D) Schematics of luciferase reporter plasmid containing 3U1 fragment of Drp1 mRNA (psiDrp1 3U1) and control plasmid (psiCHECK2). (E) Relative reporter activity was assessed by luciferase assay after transfection either siRNAs or plasmids in SH-SY5Y cells. Renilla luciferase activity was normalized with firefly luciferase activity. Data are represented as the mean ± SEM (n = 3, and * p < 0.05 value).

RNA binding proteins (RBPs) play critical roles in post-transcriptional regulation as well as in RNA metabolism, thereby regulating gene expression [30]. In order to examine the post-transcriptional regulation of Drp1 expression, we synthesized and screened a small siRNA set consisting of elucidated RBPs. Our findings revealed that hnRNP A1 regulates Drp1 expression in SH-SY5Y cells (Supplementary Fig. S1). In order to verify the screening results, we further addressed the interaction between hnRNP A1 and Drp1 mRNA in SH-SY5Y cells by ribonucleoprotein complex-immunoprecipitation (RNP-IP) assay. Consistent with the screening results, Drp1 mRNA was significantly enriched in the hnRNP A1-containing IP complex compared with in the IgG control (Fig. 1A). Since RBPs usually interact with 3′UTR of target mRNA and affect their expressions, we prepared biotinylated fragments spanning 3′UTR of Drp1 mRNA (3U1 and 3U2) and investigated interactions between hnRNP A1 and biotin-labeled 3U1/3U2 fragments by Western blotting. As shown in Fig. 1B, hnRNP A1 associated with 3U1 fragment of Drp1 mRNA but not with 3U2 fragment of negative control GAPDH, indicating that hnRNP A1 interacts directly with Drp1 mRNA via its 3′UTR (U1 region). Next, we investigated whether hnRNP A1 affects Drp1 expression by knock-down or over-expression of hnRNP A1. Although there was no significant change on the level of Drp1 mRNA by modulation of hnRNP A1 expression, the protein level of Drp1 was notably decreased by down-regulation of hnRNP A1 (Fig. 1C). Additionally, over-expression of hnRNP A1 resulted in enhanced Drp1 expression in neuroblastoma cells, suggesting that hnRNP A1 positively regulates Drp1 expression (Fig. 1C). To further investigate whether hnRNP A1 increases Drp1 expression by binding its 3′UTR region, we established a luciferase reporter plasmids harboring the 3U1 fragment of Drp1 mRNA and assessed reporter expression by luciferase activity assays after alteration of hnRNP A1 levels (Fig. 1D). Consistently, down-regulation of hnRNP A1 decreased reporter expression harboring 3U1 fragment of Drp1 mRNA, while ove- expression of hnRNP A1 enhanced luciferase activity (Fig. 1E). Taken together, these results suggest that hnRNP A1 binds to the 3′UTR of Drp1 mRNA and positively regulates Drp1 expression.

### 3.2 Down-regulation of hnRNP A1 increases mitochondrial elongation and reduces Drp1 protein level

**Fig. 2:**
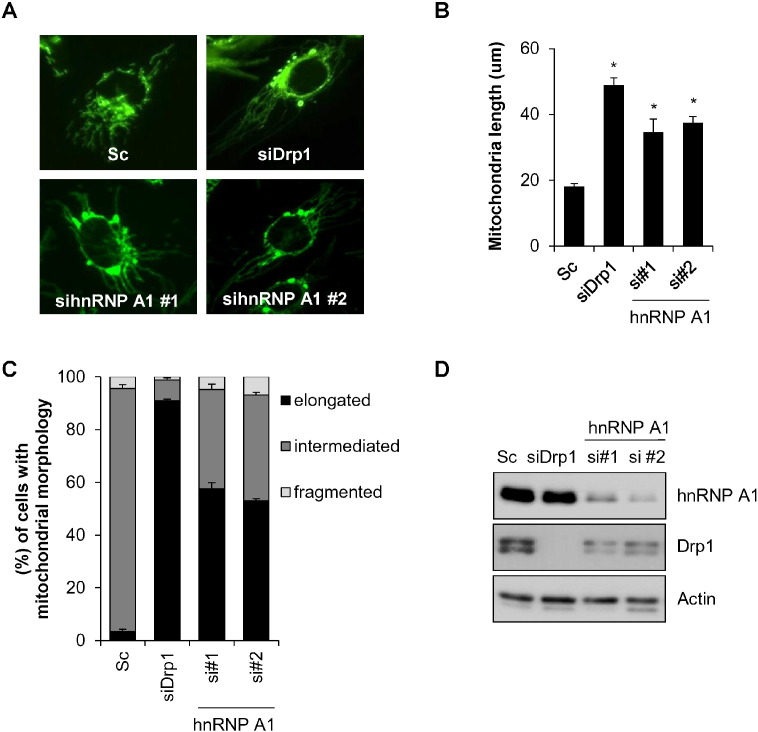
Down-regulation of hnRNP A1 induces mitochondrial elongation. SK-N-MC cells stably expressing mito-YFP (SK-N-MC/mito-YFP) were transfected with control scrambled siRNA (Sc) or siRNA against hnRNP A1 (sihnRNP A1) for 3 days. (A) Representative fluorescence pictures showing mitochondrial morphology. The siRNA for Drp1 (siDrp1) was used as a positive control. (B and C) The mitochondrial length (B) and mitochondrial morphology (C) were monitored using a fluorescence microscope. (D) The expression of hnRNP A1 (si#1, si#2) and Drp1 by their siRNA was confirmed by Western blot analysis with each antibody. Data are represented as the mean ± SEM (n > 3, and *p < 0.05 value).

Drp1 plays important roles in various mitochondrial functions such as mitochondrial morphology, motility, mitochondrial distribution and biogenesis [2]. As our data showed that hnRNP A1 binds to Drp1 mRNA, we investigated the effect of hnRNP A1 on mitochondrial dynamics. SK-N-MC neuroblastoma cells stably expressing mito-tracker fused YFP protein (SK-N-MC/mito-YFP) were transfected with siRNA against hnRNP A1 (hnRNP A1, si#1, si#2), and mitochondrial morphology was subsequently observed. In accordance with previous reports, depletion of Drp1 by siRNA was found to result in mitochondrial elongation. Interestingly, similar to what is seen with Drp1 depletion, knockdown of hnRNP A1 was also found to induce mitochondrial elongation (Fig. 2A). In addition, the length of mitochondrial filaments was significantly enhanced in cells with loss of hnRNP Al (Figs. 2B and C). The reduction of hnRNP A1 expression was confirmed by Western blotting (Fig. 2D). These results suggest that inhibition of hnRNP A1 induces mitochondrial elongation by decreasing of Drp1 expression.

### 3.3 Down-regulation of hnRNP A1 inhibits 3-NP-mediated mitochondrial dysfunction and cell death

**Fig. 3:**
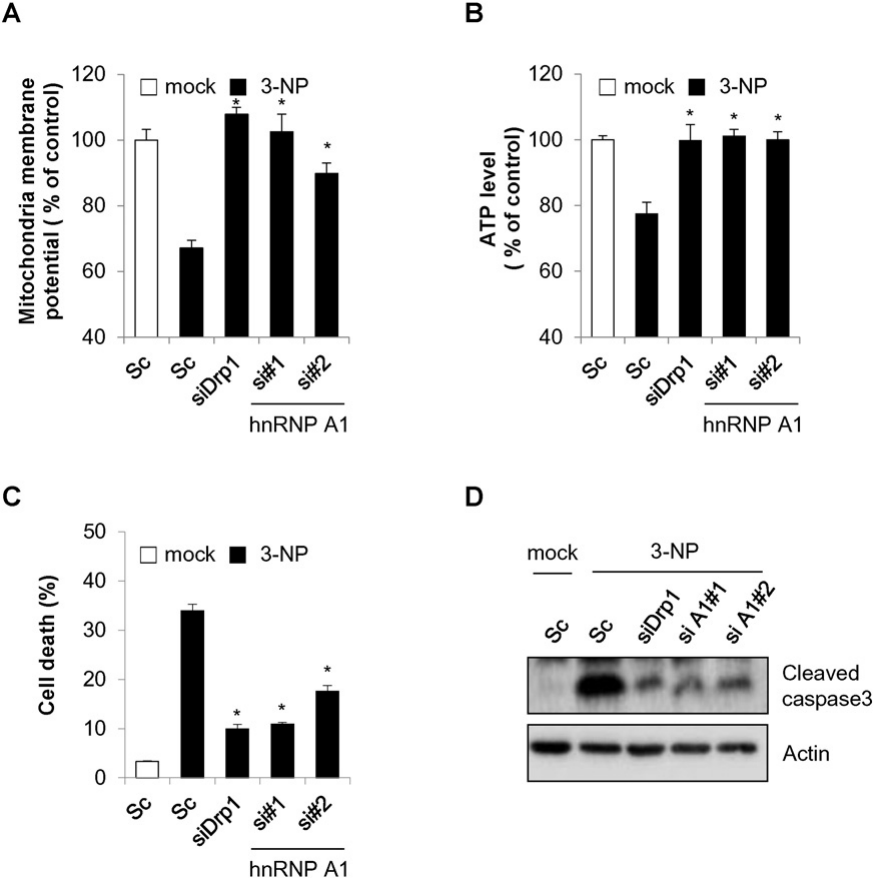
Down-regulation of hnRNP A1 inhibits 3-NP induced mitochondrial dysfunction and cell death. SK-N-MC cells were transfected with either control scrambled siRNA (Sc) or specific siRNA for hnRNP A1 (hnRNP A1 si#1, si#2) for 3 days. The cells were subjected to 3-NP (10 mM) treatment for 8 h. The siRNA for Drp1 (siDrp1) was used as a control. (A) Mitochondria membrane potential was measured using a TMRE assay system. (B) The intracellular ATP level was determined using an ATP bioluminescence assay system. (C and D) SK-N-MC cells transfected with either scrambled (Sc) or hnRNP A1 siRNA (si#1, si#2) for 3 days were treated with 3-NP (10 mM) for additional 24 h. (C) Cytotoxicity was measured by using a LDH activity assay system. (D) The cell death was assessed by Western blotting with a cleaved caspase-3 antibody. Data are represented as the mean ± SEM (n > 3, and *p < 0.05 value).

Abnormal alterations of mitochondrial morphology, which affect mitochondrial function, are related to neurodegenerative diseases such as AD and HD [6], [35]. In order to examine the effect of hnRNP A1 on mitochondrial dysfunction, SK-N-MC cells depleted hnRNP A1 or Drp1 were further treated with 3-NP, and mitochondrial function and cytotoxicity were assessed. As predicted, treatment with 3-NP induced not only disrupted mitochondrial membrane potential but also decreased cellular ATP levels (Fig. 3A and B). However, the loss of mitochondrial membrane potential and ATP levels was significantly recovered by reduction of hnRNP A1 (Fig. 3A and B). Similarly, inhibition of Drp1 also reduced mitochondrial dysfunction in 3-NP treated cells (Fig. 3A and B). Since mitochondrial dysfunction is linked to the initiation of cell death, we further examined the effect of hnRNP A1 on 3-NP-induced cytotoxicity. SK-N-MC cells transfected with siRNA for hnRNP A1 were further incubated with 3-NP and cell death and caspase activation were addressed. Both cell death and caspase-3 activation were increased after treatment with 3-NP. As with Drp1 knockdown, loss of hnRNP A1 was also fond to inhibit cell death and caspase activation in 3-NP-treated cells (Fig. 3C and D). Collectively, these findings indicate that mitochondrial elongation by hnRNP A1 depletion inhibits 3-NP-mediated mitochondrial dysfunction and cell death.

### 3.4 Over-expression of hnRNP A1 induces elevation of Drp1 protein level

**Fig. 4:**
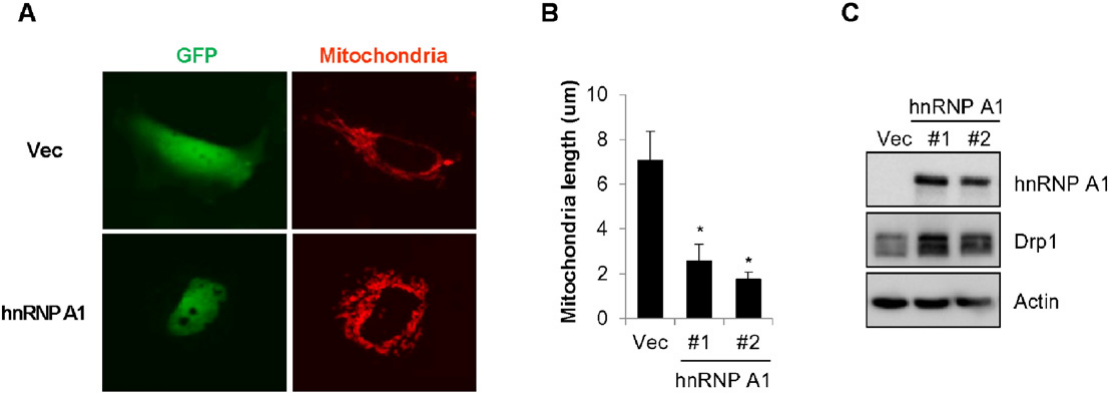
Over-expression of hnRNP A1 enhances expression of Drp1. SK-N-MC cells stably expressing control GFP (SK-N-MC/GFP) or GFP-fused-hnRNP A1 (SK-N-MC/GFP-hnRNP A1, #1, #2) were stained with a mito-tracker to image mitochondria. Then the mitochondria morphology was monitored using a fluorescence microscope. (A) Representative fluorescence images of mitochondrial morphology by over-expression of pEGFP (Vec) or pEGFP-hnRNP A1 (#1 and #2). (B) The mitochondrial length was determined by using a ‘Cell Sense Standards’ software (Olympus, Hamburg, Germany) with a fluorescence microscope. (C) The expressions of hnRNP A1 (#1, #2) and Drp1 were confirmed by Western blot analysis. Data are represented as the mean ± SEM (n > 3, and *p < 0.05 value).

Next, we examined the effect of over-expression of hnRNP A1 on mitochondrial dynamics. SK-N-MC/hnRNP A1 cells stably expressing GFP-fused-hnRNP A1 were stained with a mito-tracker dye. As shown in Fig. 4A, hnRNP A1 mainly localized to the nucleus, and the length of mitochondria was shortened in hnRNP A1 over-expressing cells (Fig. 4A and B). Then, we further investigated the Drp1 expression in hnRNP A1 over-expressing cells. Consistent with our previous data, ectopic expression of hnRNP A1 increased Drp1 expression (Fig. 4C), suggesting that over-expression of hnRNP A1 results in shortened mitochondrial length by elevating Drp1 expression in neuroblastoma cells.

### 3.5 Over-expression of hnRNP A1 potentiates 3-NP-mediated mitochondrial dysfunction and cell death

**Fig. 5:**
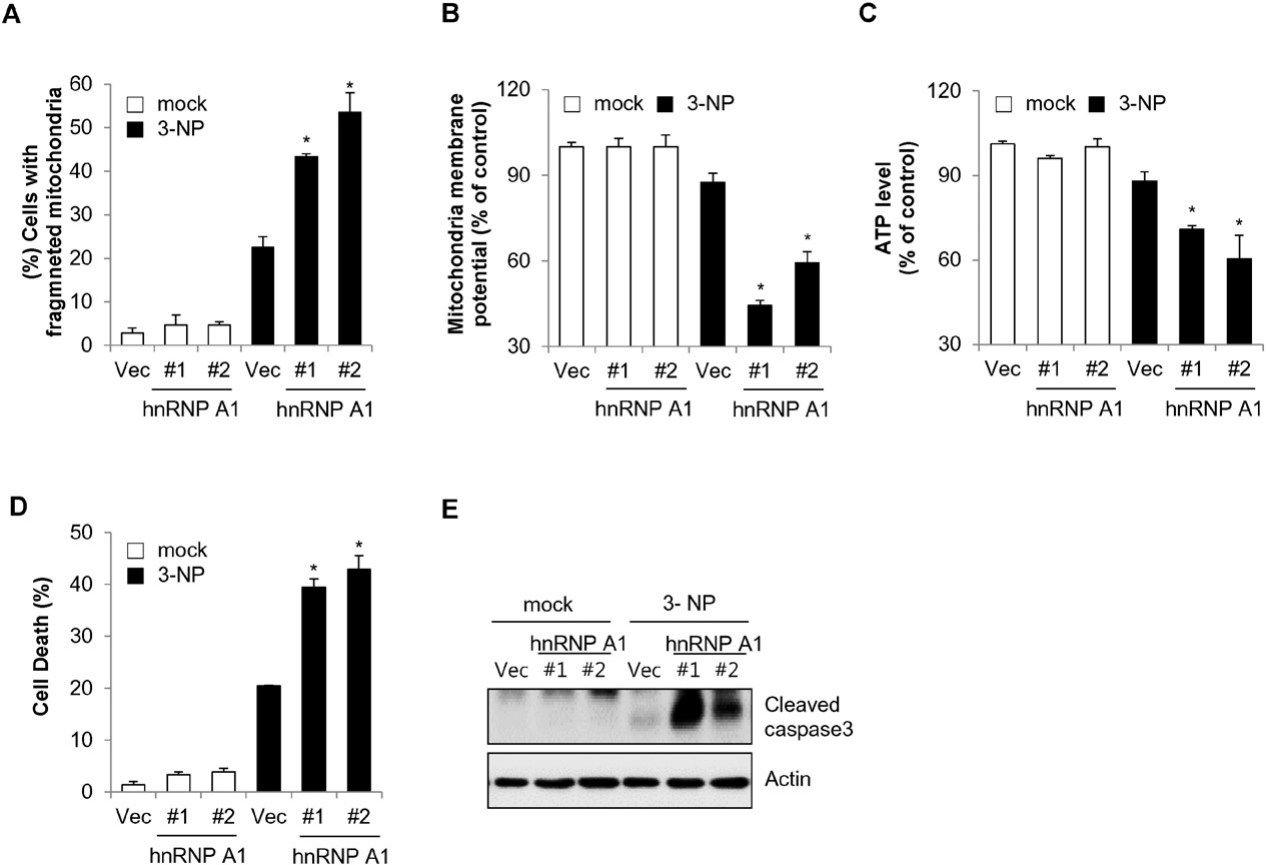
Over-expression of hnRNP A1 potentiates 3-NP-mediated mitochondrial dysfunction and cell death. SK-N-MC/GFP and SK-N-MC/GFP-hnRNP A1 cells were treated with 3-NP (10 mM) or not for 8 h. (A) Mitochondrial fragmentation was observed under a fluorescence microscope. (B and C) The mitochondrial membrane potential (B) and intracellular ATP level (C) were addressed using assay systems as described in the Materials and methods section. (D and E), SK-N-MC/GFP cells (Vec) and SK-N-MC/GFP-hnRNP A1 cells (hnRNP A1#1, and #2) were treated with 3-NP (10 mM) for 24 h. And cytotoxicity and caspase activation were analyzed by LDH activity assay kit (D) and cleaved caspase-3 antibody (E). Data are represented as the mean ± SEM (n > 3, and *p < 0.05 value).

Our findings showed that hnRNP A1-mediated mitochondrial elongation suppressed mitochondrial fragmentation as well as mitochondrial dysfunction in response to 3-NP. Therefore, we further addressed the effect of hnRNP A1 over-expression on 3-NP-mediated mitochondrial dysfunction. The mitochondrial membrane potential, total cellular ATP levels, and mitochondrial morphology were examined in SK-N-MC cells and SK-N-MC/hnRNP A1 cells treated with 3-NP. As expected, over-expression of hnRNP A1 synergistically induced mitochondrial fission in response to 3-NP treatment (Fig. 5A). Similarly, over-expression of hnRNP A1 potentiated both loss of mitochondrial membrane potential and the reduction of ATP levels in 3-NP treated cells (Fig. 5B and C). Next we examined the effect of hnRNP A1 over-expression on cell death by investigating cytotoxicity in 3-NP-treated SK-N-MC cells and SK-N-MC/hnRNP A1 cells. As shown in Fig. 5D, the ectopic expression of hnRNP A1 led to high cytotoxicity in response to 3-NP treatment in SK-N-MC cells and SK-N-MC/hnRNP A1 cells than that of control cells. Furthermore, we observed that the activation of capase-3 was also increased as a result of over-expression of hnRNP A1 and 3-NP treatment (Fig. 5E). Taken together, these results suggest that over-expression of hnRNP A1 aggravates impaired mitochondrial function and cell death in response to under 3-NP-treatment.

### 3.6 3-NP promotes translocation of hnRNP A1 to the cytoplasm and enhances Drp1 expression

**Fig. 6:**
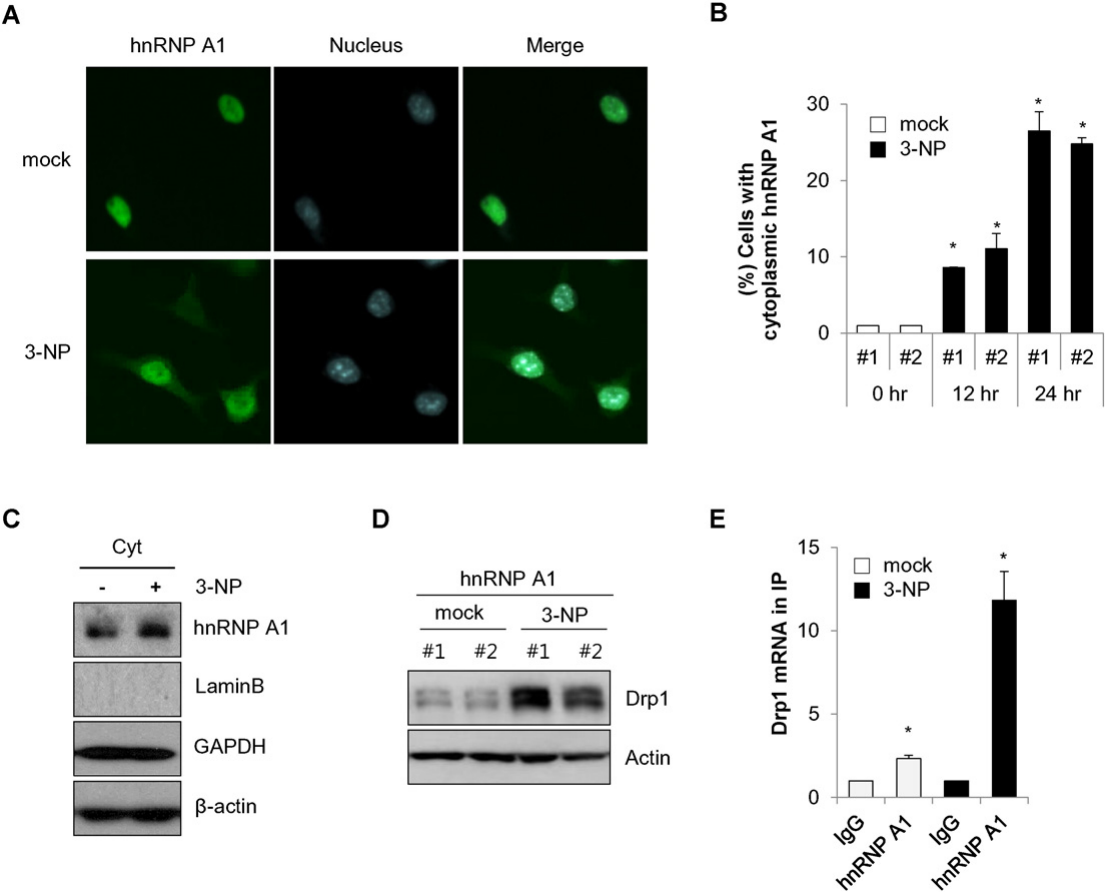
3-NP induces cytosolic translocalization of hnRNP A1 and enhances Drp1 expression. SK-N-MC/GFP-hnRNP A1 cells (#1 and #2) were incubated in the presence or absence of 3-NP (10 mM) for 16 h. (A) The cells were stained with a Hoechst dye for nuclear staining. Cytoplasmic translocation of hnRNP A1 was imaged by a fluorescence microscopy. (B) Cells showing cytoplasmic hnRNP A1 in the presence of 3-NP were counted with a fluorescence microscope. (C) Cytoplasmic RNP A1 was determined by cell fractionation and Western blotting with indicated antibodies. Both lamin B and GAPDH were used for controls. (D and E) SK-N-MC/hnRNP A1 cells (#1 and #2) exposed to 3-NP for 24 h were harvested and analyzed by Western blotting using Drp1 antibody (D). The cell lysates were subjected to RNP complex immunoprecipitation followed by RT-qPCR analysis to detect enriched Drp1 mRNA in hnRNP A1 immunoprecipitates (hnRNP A1). Data are represented as the mean ± SEM (n > 3, and *p < 0.05 value).

hnRNP A1 is predominantly found in the nucleus, however, it may be shuttled from nucleus to cytoplasm under cellular stress conditions [36], [37]. As hnRNP A1 was shown to regulate mitochondrial dynamics under cytotoxic stress conditions, we investigated the effect of cellular localization of hnRNP A1 in response to such stress conditions. SK-N-MC/hnRNP A1 cells were treated with 3-NP and the cellular location of hnRNP A1 was observed. Our results confirmed that hnRNP A1 was mainly localized in the nucleus (Fig. 6A). Interestingly, treatment with 3-NP promoted the translocation of hnRNP A1 into the cytoplasm (Fig. 6A). Cytoplasmic hnRNP A1 levels markedly increased in a time-dependent manner in 3-NP treated cells compared with those in control cells (Fig. 6B). In addition, an increase of cytoplasmic hnRNP A1 was confirmed by factionation assay (Fig. 6C), suggesting that treatment with 3-NP induces translocation of hnRNP A1 from nucleus to cytoplasm. As over-expression of hnRNP A1 increased the level of Drp1, we additionally examined the effect of hnRNP A1 on Drp1 expression in cells treated with 3-NP. Notably, treatment with 3-NP led to significant elevation of Drp1 expression in hnRNP A1 over-expressing cells (Fig. 6D). Because we observed that hnRNP A1 binds to Drp1 mRNA and regulates its expression (Fig. 1), we further examined the association between hnRNP and Drp1 mRNA after treatment with 3-NP. As shown in Fig. 6E, treatment with 3-NP makedly resulted in an increas of the interaction between hnRNP A1 and Drp1 mRNA compared with that of mock-treated contol cells (Fig. 6E). Taken together, these results indicate that 3-NP-mediated translocation of hnRNP A1 to the cytoplasm enhances Drp1 expression in neuroblastoma cells.

## 4 Discussion

Regulation of gene expression is important for multiple cellular processes such as cellular proliferation, differentiation, development, and cell death. Additionally, post-transcriptional regulation of gene expression underlies many aspects of cellular physiology as well as pathology. The two main modulators of post-transcriptional regulation are RBPs and noncoding RNAs [30], [38]. RBPs participate in each step of RNA processing, from transcription, splicing, and polyadenylation to RNA modification, transport, translation, and turnover [30]. MicroRNAs (miRNA) are small non-coding RNAs that function in gene silencing by modulating mRNA translation as well as mRNA degradation [39]. Recent studies have reported post-transcriptional regulation of mitochondrial dynamics. Wang and Li et al. showed that miR-499 and miR-30 affect mitochondrial fission machinery by targeting Drp1 [40], [41]. In addition, it was reported that HuR, a member of the ELAV family of RNA binding proteins directly binds to the 5′UTR of Bcl-xL mRNA, and that inhibition of HuR modulates mitochondrial morphology in U2OS cells [42]. However, the role of RBPs in mitochondrial dynamics is largely unknown. In the present study, we firstly showed that hnRNP A1 regulates Drp1 expression by directly binding to Drp1 mRNA.

In order to identify novel Drp1 regulatory RBPs, we generated a small-scale siRNA library set composed of 10 well-elucidated RBPs. We screened the siRNA library set and identified hnRNP A1 as a novel regulator of Drp1 expression in neuroblastoma cells (Fig. 1 and Supplementary Fig. S1). hnRNP A1, which belongs to the hnRNP subfamily, plays a role in pre-mRNA splicing, and predominantly localizes to the nucleus [43], [44]. hnRNAP A1 is also known to function as a post-transcriptional regulator, influencing mRNA turnover and translation in cytoplasm [45]. Various targets, such as the mRNAs for cyclin D1, c-Myc and interleukin-2, are post-transcriptionally regulated by hnRNP A1 [46], [47]. In the present study, we showed that hnRNP A1 affects Drp1 expression and regulates mitochondrial dynamics (Figs. 1 and 2). RBPs possess several RNA binding domains such as the RNA-recognition motif (RRM), serine/arginine-rich splicing factors (SR), K-homology domain (KH), and RGG box domain [48]. Structural analysis indicates that hnRNP A1 contains two RRM domains at the N-terminus, an RGG domain in the middle part, and a M9 domain that includes the nucleus localization sequence at the C-terminus. We showed that the 3′UTR (U1 region) of Drp1 mRNA is involved in the interaction with hnRNP A1. However, further elucidation of the specific domains of hnRNP A1 that mediate this interaction should help in understanding the biochemical mechanism underlying the regulation of Drp1 by hnRNP A1.

The disruption of mitochondrial dynamics is associated with the pathogenesis of several neurodegenerative diseases [6], [35]. HD is caused by mutations of the *Huntingtin* (*HTT*). The mutant HTT directly binds to Drp1 and increases its GTPase activity, leading to a massive mitochondrial fragmentation and neuronal damage [49]. Interestingly, it was recently reported that mitochondrial fission mediators such as Drp1 and Fis1 were increased in the post-mortem brains of HD patients, whereas mitochondrial fusion inducers such as OPA1 and Mfn1/2 were decreased [10], [50]. The 3-NP toxin when administered to rodents mimics aspects of the pattern of cell loss seen in HD [9]. 3-NP enhances reactive oxygen species (ROS) and reactive nitrogen species (RNS) which induce mitochondrial fragmentation and cell death in neuronal cells [15], [51]. Inhibition of Drp1 suppresses RNS-mediated mitochondrial fission and neuronal injury [15], [24], [51], [52]. In this study, we showed that hnRNP A1 regulates mitochondrial dysfunction and cell death in response to 3-NP (Figs. 3 and 5). Recently, it was reported that the reduced hnRNP A1 level in the brain tissues of AD patients is associated with impaired learning and memory ability [53]. However, it is still unclear whether the correlation between hnRNP A1 and mitochondrial dynamics is involved in HD or not. It could be possible that altered hnRNP A1 expression modulates mitochondrial morphology by controlling Drp1 expression in HD. Therefore, further investigation of the expression of hnRNP A1 in HD should help elucidate the role of hnRNP A1 in HD pathogenesis.

It was reported that hnRNP A1 is redistributed to the cytosol under certain stress conditions [37], [45]. Osmotic stress and ultra-violet-irradiation resulted in cytoplasmic accumulation of phosphorylated hnRNP A1 [37], [45], [54]. In addition, MNK1 acting downstream of p38 kinase, regulates the phosphorylation and subcellular distribution of hnRNP during the senescence of fibroblasts [55]. Intriguingly, we also found that treatment with the neurotoxin, 3-NP, efficiently induced translocation of hnRNP A1 from nucleus to cytoplasm, and enhanced its binding affinity to Drp1 mRNA (Fig. 6). Recently, Jang et al. showed that the phosphorylation of MAPKs such as JNK, ERK1/2, and p38, was increased in 3-NP-injected mice, and inhibition of p38 and ERK1/2 prevented neurological impairment induced by 3-NP [56]. The mechanisms underlying 3-NP-mediated translocation of hnRNP A1 need to be further elucidated in HD models, however, our results suggest that hnRNP A1 induces mitochondrial fragmentation and dysfunction by increasing Drp1 expression in 3-NP-treated cells. Since excessive mitochondrial fission and dysfunction are known to be hallmarks of neurodegenerative disease, it is necessary to elucidate the precise mechanism by which hnRNP A1 regulates Drp1 expression in various neurodegenerative diseases.

The following are the supplementary data related to this article.

Supplementary Table 1 List of primers.

Supplementary Fig. 1 Suppression of hnRNP A1 reduces Drp1 expression.
SH-SY5Y cells were transfected with either a control scrambled siRNA(Sc) or RNA binding protein specific siRNAs, including HuB, HuC, HuD, hnRNP A1, hnRNP C, hnRNP E1, hnRNP E2, hnRNP H1, hnRNP H2, and hnRNP I for 3 days. Then the expression level of Drp1 was analyzed by Western blotting.

## Transparency document

Transparency document.
