# Supplementary material for: Heterogeneous nuclear ribonucleoprotein A1 post-transcriptionally regulates Drp1 expression in neuroblastoma cells
Source: Biochim Biophys Acta. 2015 Dec;1849(12):1423–31. doi: 10.1016/j.bbagrm.2015.10.017 (PMC4655839; doi:10.1016/j.bbagrm.2015.10.017)
Supplement: Supplementary Table 1 — List of primers. [file mmc1.docx]

**Supplementary Table 1.**

| Primer name | Sequences |
| --- | --- |
| For RT-qPCR |  |
| Human GAPDH F | TGCACCACCAACTGCTTAGC |
| Human GAPDH R | GGCATGGACTGTGGTCATGAG |
| Human Drp1 F | ACCCGGAGACCTCTCATTCT |
| Human Drp1 R | TGACAACGTTGG GTGAAA AA |
| For BPD |  |
| Human Drp1-3U1-F | CCAAGCTTCTAATACGACTCACTATAGGGAGAAGAG AACTATGTAATACTGA |
| Human Drp1-3U1-R | GCC CGG CCA ATT TTG TAT |
| Human Drp1-3U2-F | CCAAGCTTCTAATACGACTCACTATAGGGAGAGGAGAAA  CCCCGT CTCTACTAA |
| Human Drp1-3U2-R | GTCTCCCTTTCTCTGGGAG |
| For Luciferase reporter |  |
| Drp1 3u1_Fwd | AAAACTCGAGTAAAGAGAACTATGTA |
| Drp1 3u1_Rev | AAAAGCGGCCGCGCCCGGCCAATTT |
