# Supplementary figures and images for: Heterogeneous nuclear ribonucleoprotein A1 post-transcriptionally regulates Drp1 expression in neuroblastoma cells
Source: Biochim Biophys Acta. 2015 Dec;1849(12):1423–31. doi: 10.1016/j.bbagrm.2015.10.017 (PMC4655839; doi:10.1016/j.bbagrm.2015.10.017)

## Slide 1
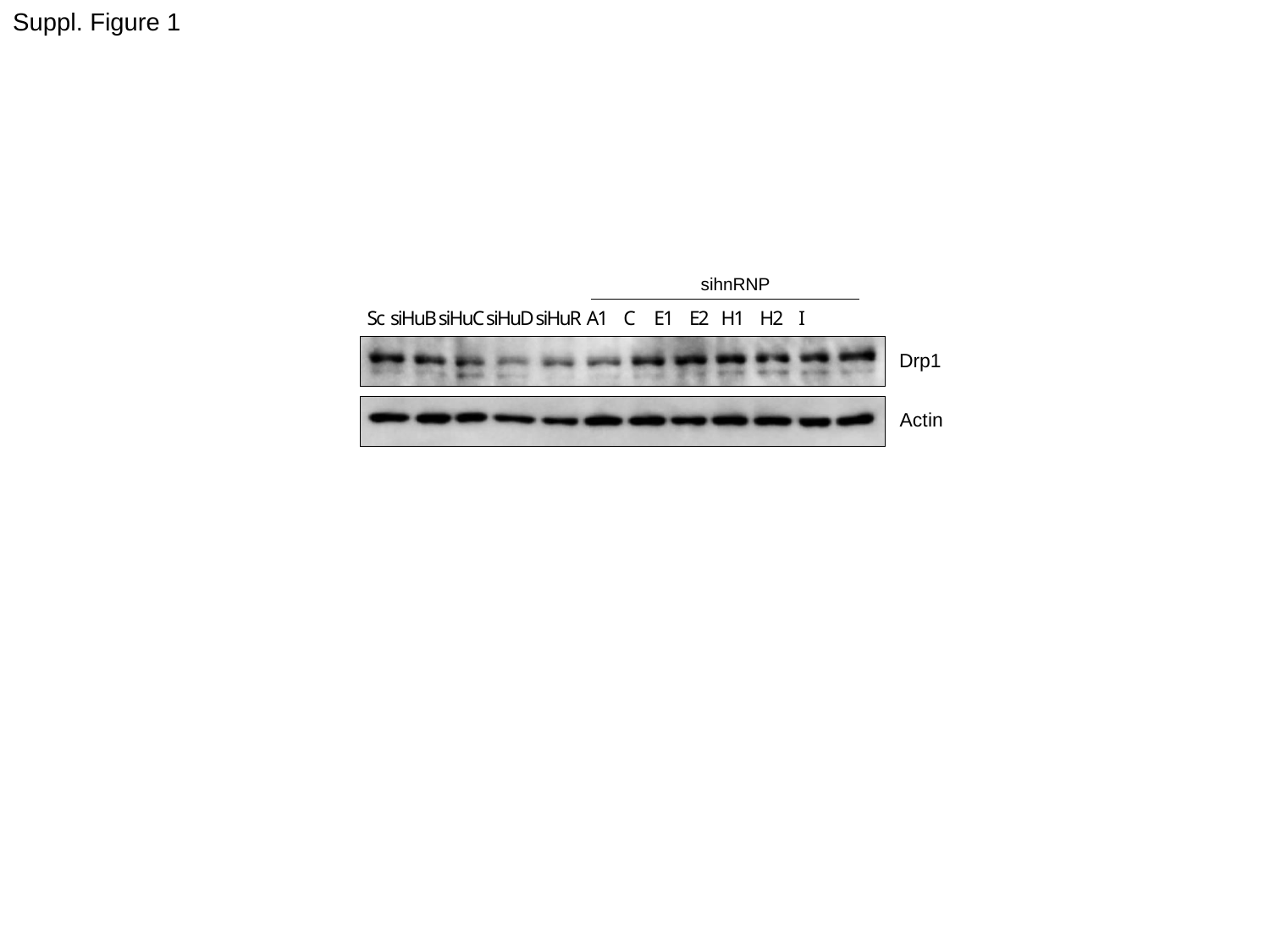

Suppl. Figure 1
sihnRNP
 Sc siHuB siHuC siHuD siHuR A1 C E1 E2 H1 H2 I
Drp1
Actin

Supplement: Supplementary Fig. 1 — Suppression of hnRNP A1 reduces Drp1 expression. SH-SY5Y cells were transfected with either a control scrambled siRNA(Sc) or RNA binding protein specific siRNAs, including HuB, HuC, HuD, hnRNP A1, hnRNP C, hnRNP E1, hnRNP E2, hnRNP H1, hnRNP H2, and hnRNP I for 3 days. Then the expression level of Drp1 was analyzed by Western blotting. [file mmc2.pptx]
